# Effectiveness and Safety of Ozone Therapy in Humans: An Umbrella Review of Systematic Reviews with Meta-Analyses of Randomized Clinical Trials

**DOI:** 10.3390/medsci14020289

**Published:** 2026-06-04

**Authors:** Stefano Cacciatore, Gabriele Abbatecola, Riccardo Calvani, Nicola Veronese

**Affiliations:** 1 Department of Physiology and Aging, College of Medicine, University of Florida, Gainesville, FL 32611, USA; 2 Department of Geriatrics, Orthopedics and Rheumatology, Università Cattolica del Sacro Cuore, 00168 Rome, Italy; 3 Fondazione Policlinico Universitario “Agostino Gemelli” IRCCS, 00168 Rome, Italy; 4 Faculty of Medicine, Saint Camillus International University of Health Sciences, 00131 Rome, Italy

**Keywords:** ozone therapy, periodontitis therapy, diabetic foot therapy, COVID-19 therapy, impacted tooth surgery, evidence-based medicine

## Abstract

Background/Objectives: Ozone therapy has been proposed across multiple clinical conditions based on hormetic, antioxidant, and immunomodulatory effects, but its efficacy and safety remain controversial. We conducted an umbrella review to appraise the effectiveness and safety of ozone therapy using evidence from meta-analyses of randomized controlled trials (RCTs). Methods: We searched MEDLINE, Web of Science, Embase, and the Cochrane Library from inception to 14 February 2025, with an updated search performed on 9 May 2026. Eligible studies were systematic reviews with meta-analyses comparing ozone therapy with non-active controls, including placebo, sham, saline, or standard care. Methodological quality was evaluated with AMSTAR-2 and certainty of evidence with GRADE. Results: Of 1243 records identified, seven meta-analyses representing four clinical indications (chronic periodontitis, COVID-19, diabetic foot ulcers, and impacted mandibular third-molar surgery) were included. In chronic periodontitis, evidence was mixed: one meta-analysis found no significant adjunctive benefit, whereas a more recent meta-analysis reported improvements in probing depth and gingival index, but not in bleeding on probing, plaque index, or clinical attachment level. For COVID-19, ozone therapy reduced PCR positivity at follow-up (RR 0.07; 95% CI 0.01–0.34), although this was considered a clinically non-important surrogate endpoint, and showed no significant benefit for hospital stay, intensive care unit admission, or mortality. For diabetic foot ulcers, ozone therapy was not superior to control treatment for ulcer healing (RR 1.69; 95% CI 0.90–3.17) or reduction in ulcer area. In third-molar surgery, ozone therapy did not reduce swelling or improve mouth opening, but was associated with improved short-term quality of life and reduced analgesic use. Safety outcomes were inconsistently reported, and available data did not allow firm conclusions regarding adverse events. The certainty of evidence was low or very low for all outcomes. Conclusions: Despite mechanistic plausibility, current meta-analytic evidence from RCTs remains inconsistent, methodologically fragile, and largely based on low- or very low-certainty findings. Routine clinical use is not justified pending adequately powered, blinded RCTs with standardized dosing and delivery, patient-centered endpoints, and rigorous safety monitoring.

## 1. Introduction

Ozone therapy is a medical treatment based on the controlled administration of ozone gas for therapeutic purposes. In recent decades, it has received increasing attention in both clinical and research settings [1]. Initially explored for its potent antimicrobial and disinfectant properties, ozone was primarily employed in the context of infection control. Over time, however, its use has progressively expanded to a broader range of medical conditions [1]. Ozone therapy is thought to trigger a cascade of biological responses primarily through the induction of a controlled, transient oxidative stimulus. This process leads to the formation of reactive oxygen species, most notably hydrogen peroxide, and lipid ozonation products, which in turn activate cellular antioxidant pathways. Among these, the nuclear factor erythroid 2-related factor 2 (Nrf2)-mediated transcription of antioxidant response elements plays a central role, enhancing the production of enzymes such as superoxide dismutase, glutathione peroxidase, and catalase. These mechanisms are believed not only to contribute to redox homeostasis but also to improved tissue oxygenation and immunomodulation [2]. Its anti-inflammatory and analgesic potential has been particularly highlighted in the context of chronic musculoskeletal pain and degenerative joint disorders [3]. Furthermore, ozone has been proposed as adjunctive treatment in infectious diseases [4], including COVID-19 [5], vascular disorders [6], and wound healing [7]. Despite these promising observations, however, ozone remains a controversial intervention, with its scientific credibility challenged by inconsistent trial outcomes, heterogeneous methodologies, and possible safety concerns [7]. Regulatory bodies such as the U.S. Food and Drug Administration have reiterated that ozone is a toxic gas with no established role in medicine [8]. Moreover, systematic reviews and meta-analyses have frequently highlighted the limited quality of available evidence and the lack of robust intervention studies [9]. In this context of uncertainty and fragmented evidence, a comprehensive synthesis is warranted to evaluate both the efficacy and safety of ozone therapy.

This umbrella review aims to systematically appraise evidence from meta-analyses of randomized controlled trials investigating ozone therapy in human subjects across a range of clinical indications. Specific objectives include: (1) assessing the efficacy of ozone therapy in comparison to non-active controls such as placebo, saline, or standard care, with a focus on clinically relevant outcomes; (2) evaluating the safety profile of ozone therapy based on the frequency and severity of reported adverse events; and (3) determining the credibility and robustness of the available evidence using the Grading of Recommendations Assessment, Development and Evaluation (GRADE) framework [10].

## 2. Materials and Methods

This umbrella review of systematic reviews with meta-analyses was conducted in accordance with the methodological guidance of the Cochrane Handbook for Systematic Reviews of Interventions [11], and reported following the updated 2020 Preferred Reporting Items for Systematic Reviews and Meta-Analyses (PRISMA) guidelines [12]. The study protocol was registered in the Open Science Framework on 1 October 2025 (https://osf.io/sbujx/) database and is publicly accessible.

### 2.1. PICO Question and Eligibility Criteria

Following the PICOS framework, we included studies enrolling human participants with any medical condition and evaluating ozone therapy, defined as the administration of medical ozone in any form, including systemic administration, intra-articular injection, rectal insufflation, or topical application, with therapeutic intent. Eligible comparators were non-active controls, including placebo, saline, sham treatment, or standard care. Outcomes of interest included clinical efficacy endpoints relevant to the treated condition, as well as safety endpoints, such as the incidence and severity of adverse events. In terms of study design, we included systematic reviews with meta-analyses of randomized clinical trials (RCTs).

We excluded the following studies: (i) systematic reviews without meta-analyses; (ii) reviews based exclusively on observational or non-randomized studies; (iii) reviews evaluating ozone therapy in combination with other active treatments when separate effect estimates for ozone were not available.

### 2.2. Information Sources and Search Strategies

We conducted a comprehensive literature search in four electronic databases: MEDLINE (via PubMed), Web of Science, Embase, and the Cochrane Library, from inception to 14 February 2025. The search was subsequently updated on 9 May 2026, using the same search strategy to identify newly published eligible systematic reviews and meta-analyses. The search strategy combined controlled vocabulary (e.g., MeSH terms) and free-text keywords related to “ozone therapy”, “systematic review”, “meta-analysis”, and “randomized controlled trial”. Database-specific strategies were adapted to the syntax of each source and are reported in detail in Supplementary Table S1. In addition, reference lists of all eligible articles were manually screened to identify any further potentially relevant reviews not retrieved through the initial search.

### 2.3. Study Selection

The selection process was independently carried out by four review authors (S.C., G.A., R.C., N.V.) using the Covidence software platform (Covidence systematic review software, Veritas Health Innovation, Melbourne, Australia. Available at www.covidence.org). Titles and abstracts identified through the systematic search were screened for potential eligibility, followed by full-text evaluation of the records that passed the initial screening. Disagreements occurring at either stage were resolved by consensus, with final decisions arbitrated by a senior reviewer (N.V.) when necessary. In cases where multiple systematic reviews addressed the same clinical question using overlapping primary studies, all eligible reviews were described and methodologically appraised. However, for the outcome-level evidence synthesis and GRADE assessment, the most comprehensive and up-to-date review, defined as the one including the highest number of eligible RCTs and the most recent search, was retained to avoid double counting of primary studies.

### 2.4. Data Collection and Data Items

Data extraction was independently performed by three reviewers (G.A., R.C., and S.C.) using a standardized and pre-piloted data extraction form. For each included systematic review, the following information was collected: first author, year of publication, journal, number of included randomized controlled trials, and total sample size. Additional data included details of ozone therapy administration (e.g., dosage, frequency, and route), comparator characteristics (placebo, saline, or standard care), health outcomes, and summary effect estimates (such as risk ratios, mean differences, and 95% confidence intervals). Where reported, data on adverse events and other safety outcomes were also extracted. Disagreements were resolved by consensus among the extractors. In case of persistent discrepancies, the final decision was made by a senior reviewer (N.V.).

### 2.5. Data Synthesis

Data were extracted as pooled estimates from the original meta-analyses. When one parameter considered for the GRADE (e.g., publication bias) was not reported, we considered this domain at potential high risk of bias.

### 2.6. Risk of Bias and Quality Assessment

The methodological quality of each included systematic review was assessed using the A MeaSurement Tool to Assess Systematic Reviews 2 (AMSTAR-2) instrument [13]. AMSTAR-2 consists of 16 items, of which seven are considered critical domains, including protocol registration, adequacy of the literature search, justification for study exclusions, and risk of bias assessment of individual studies. Each item is rated as “yes”, “partial yes”, or “no”, and the overall confidence in the results of a review is categorized into one of four levels: “high”, “moderate”, “low”, or “critically low”. Reviews are rated as “critically low” when they present more than one critical flaw, while “high” confidence is reserved for reviews with no or only one non-critical weakness [13]. In addition, the overall certainty of the evidence for each meta-analytic outcome was evaluated using the GRADE framework [10]. This approach considers five domains, i.e., risk of bias, inconsistency, indirectness, imprecision, and publication bias, to classify the quality of evidence as “high”, “moderate”, “low”, or “very low”.

## 3. Results

### 3.1. Literature Search and Study Selection

**Figure 1:**
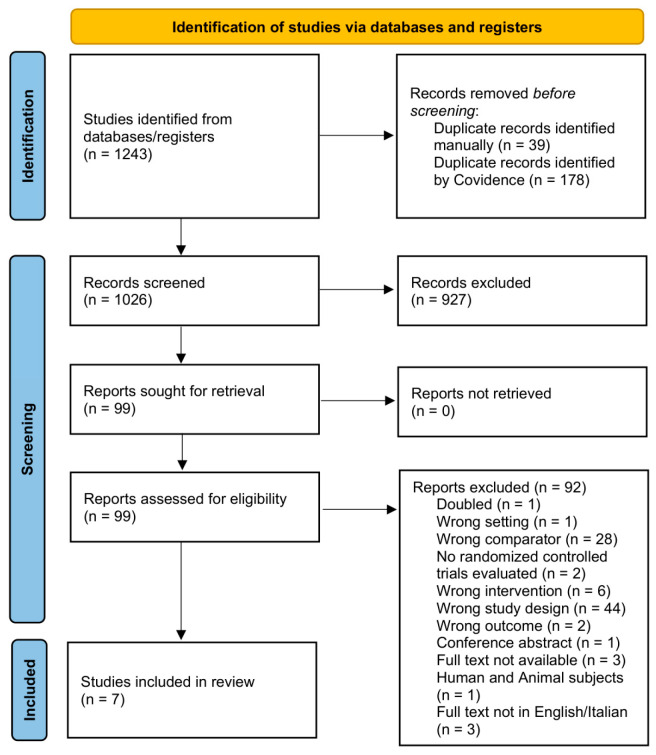
PRISMA flow diagram of the literature search and study selection process.

Figure 1 reports the literature search process for this umbrella review. Overall, we initially considered 1243 articles from which 217 duplicate records were removed before screening. Of the 1026 articles screened, we analyzed the full text of 99 reports for eligibility. Of them, 92 reports were excluded, most commonly because of wrong study design, followed by meta-analyses reporting wrong comparators (e.g., active controls such as corticosteroids). The details of the exclusion at the full-text level are reported in Supplementary Table S2. Therefore, we finally included seven meta-analyses.

### 3.2. Characteristics of Included Reviews

Table 1 reports the data about the descriptive findings of the seven meta-analyses, including the routes of administration and the dose of ozone therapy. Overall, the seven meta-analyses included four different populations, i.e., chronic periodontitis, patients hospitalized for COVID-19, diabetic foot ulcers, and patients who underwent surgery for impacted mandibular third molar.

### 3.3. Main Findings

#### 3.3.1. Ozone Therapy in Chronic Periodontitis

For chronic periodontitis, five outcome estimates from the most comprehensive and up-to-date eligible meta-analysis were reported in Table A1. Although two eligible meta-analyses addressed this indication, they partially overlapped at the level of primary randomized trials; therefore, in accordance with our prespecified approach, the most comprehensive and recent review was retained for the outcome-level GRADE synthesis to avoid double counting. The more recent meta-analysis by Liu et al. [16] suggested statistically significant improvements in probing depth and gingival index with ozone-assisted scaling and root planing, but found no significant differences for bleeding on probing, plaque index, or clinical attachment level. According to the GRADE framework, the certainty of evidence was very low for two outcomes and low for three outcomes.

#### 3.3.2. Ozone Therapy in COVID-19

Table A2 summarizes the findings on ozone therapy in patients hospitalized for COVID-19. In two RCTs, ozone therapy was associated with a lower rate of PCR positivity at follow-up compared with control treatment (RR 0.07; 95% CI 0.01–0.34), with low-certainty evidence. However, this outcome was considered not clinically important. Ozone therapy was not significantly superior to control treatment for length of hospital stay (MD 0.70 days lower; 95% CI 4.10 lower to 2.70 higher), intensive care unit admission (RR 0.44; 95% CI 0.15–1.33), or mortality (OR 0.73; 95% CI 0.32–1.68). The certainty of evidence was very low for length of hospital stay and low for intensive care unit admission and mortality.

#### 3.3.3. Ozone Therapy in Diabetic Foot Ulcers

Table A3 summarizes the findings on ozone therapy for the treatment of diabetic foot ulcers. In two RCTs including 111 patients, ozone therapy was not significantly superior to control treatment in achieving ulcer healing (RR 1.69; 95% CI 0.90–3.17), with low-certainty evidence according to GRADE. No significant benefit was observed for reduction in ulcer area (MD 2.11 lower; 95% CI 5.29 lower to 1.07 higher), which was supported by very low-certainty evidence.

#### 3.3.4. Ozone Therapy in Impacted Third-Molar Surgery

Table A4 shows the findings for patients undergoing surgery for impacted mandibular third molars. Nine outcomes were reported. Ozone therapy was not superior to control treatment in reducing swelling at 72 h or 7 days. Similar findings were observed for mouth opening at 24 h, 72 h, and 7 days. Conversely, ozone therapy was associated with improved postoperative quality-of-life scores at 24 h, 72 h, and 7 days, and with a lower number of analgesics used. No formal minimal clinically important difference was available for the quality-of-life outcomes; therefore, this isolated patient-reported signal was interpreted as being of uncertain clinical relevance. All outcomes were supported by very low-certainty evidence according to GRADE.

#### 3.3.5. Safety Profile Across Indications

Evidence on safety was inconsistently reported across the included systematic reviews and meta-analyses. The Cochrane review on diabetic foot ulcers provided the only explicit pooled safety analysis [7]. In one of the three included RCTs, no side effects were observed; in another, adverse events such as osteomyelitis, fever, wound infection, and pulmonary congestion were reported, but were not judged to be related to the study interventions. Pooled analysis did not show a significant difference between ozone therapy and control groups in terms of adverse events (RR 2.27; 95% CI 0.48–10.79). Liu et al. [16] stated that ozone therapy combined with scaling and root planing did not increase adverse reactions; however, this review did not provide a dedicated pooled safety analysis, and its quantitative synthesis focused on periodontal efficacy outcomes. Other included reviews did not report formal safety outcomes; nonetheless, no major complications emerged from the included trials, and ozone therapy was generally described as well tolerated.

### 3.4. Methodological Quality of Included Meta-Analyses

The methodological quality of the seven included meta-analyses, assessed using the AMSTAR-2 tool, is displayed in Supplementary Table S3. Two meta-analyses [7,19] achieved a high confidence rating, fulfilling most of the critical domains, including a priori protocol definition, adequate assessment of risk of bias in primary trials, and appropriate meta-analytic methods. Three reviews [15,17,18] were rated as low confidence, primarily due to shortcomings in protocol registration, reporting of excluded studies, reporting of funding sources for primary studies, assessment of the impact of risk of bias, and publication bias assessment. Two reviews [14,16] were rated as critically low because of multiple critical methodological limitations.

## 4. Discussion

In this umbrella review of meta-analyses of RCTs, we systematically evaluated the evidence regarding the efficacy and safety of ozone therapy across diverse clinical contexts. Overall, the results suggest that ozone therapy does not provide consistent benefits over placebo or standard care. In chronic periodontitis, evidence was mixed, with one meta-analysis showing no significant adjunctive benefit and a more recent meta-analysis reporting improvements limited to probing depth and gingival index, but not to bleeding on probing, plaque index, or clinical attachment level. No clear superiority was observed for diabetic foot ulcer healing or for most postoperative outcomes following third-molar surgery. Limited signals of potential benefit were reported for selected periodontal indices, patient-reported quality of life after oral surgery, analgesic use, and PCR positivity among COVID-19 patients. However, PCR positivity represents a surrogate and clinically non-meaningful endpoint [20,21], and most findings were derived from small and methodologically weak trials, with certainty of evidence rated as low or very low. Evidence on the safety profile of ozone therapy was inconsistently addressed by the included studies. The AMSTAR-2 appraisal highlighted that only two meta-analyses reached a high confidence rating, whereas the majority were classified as low or critically low confidence. Taken together, these findings delineate a body of evidence that is heterogeneous in methods and fragile in certainty, thereby tempering any claim of clinical benefit.

Several converging factors likely explain the inconsistency of the observed effects. The included studies displayed substantial heterogeneity in ozone administration routes, dosages, and treatment schedules, which hampers comparability and synthesis. Many trials were underpowered, often enrolling fewer than 100 participants, and focused on short-term outcomes. Moreover, several outcomes, such as pain relief and quality of life, are highly subjective and particularly vulnerable to placebo effects, especially where blinding was suboptimal. These issues likely contributed to inflated effect estimates in some contexts and to the overall low credibility of the observed signals of benefit [22].

Evidence in chronic periodontitis was inconsistent across the two eligible meta-analyses. Moraschini et al. [15] did not support the adjunctive use of ozone therapy in non-surgical periodontal treatment, whereas Liu et al. [16] reported statistically significant improvements in probing depth and gingival index. However, these apparent benefits were not consistent across other periodontal outcomes, including bleeding on probing, plaque index, and clinical attachment level. The clinical relevance of these modest changes remains uncertain, particularly in the context of low or very low certainty of evidence and substantial heterogeneity in ozone delivery, treatment schedules, and follow-up duration. Therefore, the updated evidence suggests a possible signal for selected surrogate periodontal parameters, but does not provide robust support for routine clinical use.

In patients with diabetic foot ulcers, the available evidence does not demonstrate a clear benefit of ozone therapy on definitive healing outcomes. In the Cochrane review [7], ozone therapy was not significantly superior to control treatment in achieving ulcer healing, and no significant benefit was observed for reduction in ulcer area. Although more recent evidence syntheses have suggested possible improvements in selected intermediate outcomes, such as wound-size reduction or hospitalization time, these findings derive from small, heterogeneous studies and, in some cases, reviews with important methodological limitations. Overall, the evidence remains insufficient to support a consistent effect of ozone therapy on patient-important wound outcomes.

For COVID-19, the evidence base remains immature and fragmented. Some meta-analyses report reductions in mortality or length of stay [17], whereas others emphasize benefits confined to surrogate markers, with no robust effect on ICU admission or other hard outcomes [19]. Given these uncertainties on efficacy, safety considerations warrant careful scrutiny.

Previous systematic reviews and meta-analyses have consistently highlighted the heterogeneity and methodological limitations of the available trials, with small sample sizes, short follow-up, and lack of standardized outcomes. While most studies focused on clinical efficacy, the issue of safety has been less systematically addressed. The included Cochrane review on diabetic foot ulcers [7] included three RCTs and reported no adverse effects in one study and a small number of complications (osteomyelitis, fever, wound infection, pulmonary congestion) in another, none of which were considered directly related to ozone therapy. Pooled analysis did not demonstrate a significant increase in adverse events compared with controls. In the context of nonsurgical periodontal therapy, no systematic reporting of adverse events was available, although the treatment was generally described as well tolerated. Similarly, the meta-analysis on COVID-19 did not include safety as a formal endpoint, but no major complications were documented, and ozone therapy was described as safe in the short term. Taken together, these findings suggest that ozone therapy appears to be well tolerated in the short term, but the quality of evidence is very low, and systematic safety monitoring is largely absent. Rare but potentially serious adverse events (including stroke, myocardial infarction, thrombosis, and hemolysis) have been described in the literature, and long-term safety data are lacking [23,24,25]. Therefore, any clinical application of ozone should be considered with caution, especially outside of controlled research settings. Future trials should include standardized assessment and reporting of adverse events as a predefined outcome, with adequate follow-up to capture possible delayed toxicities.

Mechanistic work supports a hormetic model wherein low-dose ozone induces ROS-mediated signaling and Nrf2/Keap1/ARE pathway activation, up-regulating endogenous antioxidant defenses and modulating inflammatory cascades [26,27,28,29]. While biologically this model may be coherent, translation to durable clinical benefit has been inconsistent, likely reflecting variability in dosing, delivery, and patient selection. Unsurprisingly, this mismatch between mechanistic promise and clinical proof is mirrored in current regulatory positions.

Regulatory positions should be interpreted within this broader evidentiary context. The U.S. Food and Drug Administration has stated that ozone is a toxic gas with no established medical role [8], a position that reflects concerns regarding both efficacy and safety rather than serving as a substitute for evidence appraisal. At the same time, ozone therapy continues to be used off-label or more extensively in some countries, highlighting the gap between clinical uptake and the availability of robust trial evidence. In this regard, patterns of use should be viewed as a stimulus for rigorous investigation rather than as proof of clinical efficacy. The pandemic era further amplified this tension, with rapid interest in ozone-based interventions occurring in a context of limited, heterogeneous, and often methodologically fragile evidence [30].

From a pragmatic perspective, the present synthesis does not justify routine clinical use. Future research should focus on adequately powered, blinded RCTs with standardized dosing and delivery, core outcome sets prioritizing patient-important endpoints, and prespecified safety monitoring. Evidence syntheses should employ registered protocols, comprehensive searches, transparent lists of excluded studies, and formal assessments of publication bias.

Limitations of this umbrella review include: (i) reliance on published meta-analyses of RCTs without access to individual participant data, which precluded harmonization of outcomes and subgroup/interaction analyses; (ii) potential omission of very recent single randomized trials not yet incorporated into meta-analyses (time-lag bias); (iii) substantial heterogeneity in ozone formulations, delivery routes, dosing ranges, and co-interventions across the source literature, which constrains comparability and generalizability; and (iv) limited quality of the systematic reviews included.

## 5. Conclusions

Despite mechanistic plausibility and sporadic signals on intermediate endpoints, the currently available meta-analytic evidence from randomized controlled trials remains inconsistent, methodologically fragile, and largely based on low- or very low-certainty findings. Accordingly, the routine clinical use of ozone therapy is not justified on the basis of the available evidence. Future studies should employ standardized dosing and delivery, adequately powered and blinded RCTs, patient-centered endpoints, and prespecified safety monitoring to establish, or refute, clinical utility.

## Figures and Tables

**Table 1:** Descriptive findings of the included systematic reviews and meta-analyses.

Author, Year	Type of Population	Dose Ozone Therapy	Route of Administration
Chaudhry et al., 2021 [14]	Patients undergoing surgery for impacted mandibular third molars	Not standardized across trials	Local postoperative application; 2 studies used an extraoral ozone probe, 1 used ozonated water irrigation, and 1 used topical ozone gel
Liu et al., 2015 [7]	Patients with diabetes mellitus complicated by foot ulcers	Not standardized across trials; where reported, Zhang et al. used 52 µg/mL, while other trials used device-based or local/rectal ozone protocols without a unified dose	Local or regional ozone application, including gas applied to the wound, device-based ozone-oxygen therapy, and local plus rectal insufflation depending on the included trial
Moraschini et al., 2020 [15]	Patients with chronic periodontitis undergoing nonsurgical periodontal treatment	Variable across trials; ozonated water or gaseous ozone with non-uniform concentrations and treatment schedules	Topical periodontal application, mainly sulcus irrigation with ozonated water or insertion/application of ozonated gas after scaling and root planing
Liu et al., 2025 [16]	Patients with chronic periodontitis undergoing scaling and root planing	Variable across trials; ozonated water concentrations ranged from 5–20 µg/mL to 75–85 µg/mL in some studies, while gaseous ozone protocols used variable concentrations, including 2100 ppm or 75 mg/mL	Local adjunctive periodontal therapy; ozonated water irrigation/rinsing, ozone nanobubble water, ozonized olive oil mouthwash, or gaseous ozone
Shang et al., 2023 [17]	Hospitalized adult patients with COVID-19	Variable across trials; examples include major autohemotherapy at 30–40 µg/mL, intra-rectal ozone around 35–40 µg/mL, and nebulized ozone at 0.2 ppm	Major autohemotherapy, intra-rectal insufflation, intra-rectal insufflation plus autohemotherapy, and nebulization/inhalation, depending on the included trial
Filho et al., 2024 [18]	Patients with diabetic foot ulcers	Not standardized; ozone dose, duration, and treatment modality varied substantially across studies	Local, systemic, or combined ozone therapy, including topical/local wound application and systemic administration depending on the included trial
Jafari-Oori et al., 2022 [19]	Hospitalized patients with COVID-19, with variable disease severity	Variable across studies; examples include rectal insufflation at 40 µg/mL, 150 mL twice daily, minor autohemotherapy with 5 mL ozone at 25 µg/mL, major autohemotherapy around 30–40 µg/mL, and ozone inhalation at 0.2 ppm	Major autohemotherapy, rectal insufflation, rectal insufflation plus minor autohemotherapy, and ozone inhalation/nebulization

## Data Availability

No new data were created or analyzed in this study.

## Supplementary Materials

The following supporting information can be downloaded at: https://www.mdpi.com/article/10.3390/medsci14020289/s1. Table S1: Database-specific search strategies used for the identification of eligible studies. Table S2: List of excluded studies with reasons for exclusion at full-text screening. Table S3: AMSTAR 2 quality assessment of included systematic reviews and meta-analyses.

## Appendix A

**Table A1:** Effect of ozone therapy in chronic periodontitis.

Quality Assessment							No of Patients		Effect		Quality	Importance
No of Studies	Design	RoB	Inconsistency	Indirectness	Imprecision	Other Considerations	Ozone Therapy	Control	Relative (95% CI)	Absolute		
Probing pocket depth												
12	Randomized trials	Serious ^1^	Very serious ^2^	No serious indirectness	Serious ^3^	No publication bias detected ^4^	298	297	-	WMD 0.26 lower, 95% CI 0.48 lower to 0.05 lower	VERY LOW	Critical
Gingival index												
7	Randomized trials	Serious ^1^	No serious inconsistency	No serious indirectness	Serious ^3^	None	147	147	-	WMD 0.15 lower, 95% CI 0.24 lower to 0.07 lower	LOW	Important
Bleeding on probing												
6	Randomized trials	Serious ^1^	No serious inconsistency	No serious indirectness	Serious ^5^	None	157	159	-	WMD 3.29 lower, 95% CI 8.65 lower to 2.06 higher	LOW	Important
Plaque index												
7	Randomized trials	Serious ^1^	No serious inconsistency	No serious indirectness	Serious ^5^	None	127	127	-	WMD 0.05 lower, 95% CI 0.15 lower to 0.04 higher	LOW	Important
Clinical attachment level												
9	Randomized trials	Serious ^1^	Serious ^6^	No serious indirectness	Serious ^5^	None	220	219	-	WMD 0.27 lower, 95% CI 0.56 lower to 0.01 higher	VERY LOW	Critical

1—Serious risk of bias because several trials had unclear randomization and/or allocation concealment. 2—I^2^ > 75%, indicating very serious inconsistency. 3—Serious imprecision because, despite statistical significance, the effect size was small and the total sample size remained limited for a definitive conclusion. 4—In Liu et al., publication bias was assessed for probing depth; Egger’s test did not suggest significant publication bias, *p* = 0.169. 5—Serious imprecision because the confidence interval crossed the line of no effect. 6—I^2^ = 72%, indicating serious inconsistency. Abbreviations: CI, confidence interval; RoB, risk of bias; WMD, weighted mean difference.

**Table A2:** Effect of ozone therapy in patients hospitalized for COVID-19.

Quality Assessment							No of Patients		Effect		Quality	Importance
No of Studies	Design	RoB	Inconsistency	Indirectness	Imprecision	Other Considerations	Ozone Therapy	Control	Relative (95% CI)	Absolute		
PCR positivity at follow-up												
2	Randomized trials	No serious risk of bias	No serious inconsistency	No serious indirectness	Very serious ^1^	None	1/45 (2.2%)	21/45 (46.7%)	RR 0.07 (0.01 to 0.34)	434 fewer per 1000, from 308 fewer to 462 fewer	LOW	Not important
Length of hospital stay												
2	Randomized trials	No serious risk of bias	Very serious ^2^	No serious indirectness	Very serious ^1^	None	63	59	-	MD 0.70 days lower, 95% CI 4.10 lower to 2.70 higher	VERY LOW	Important
Intensive care unit admission												
3	Randomized trials	No serious risk of bias	No serious inconsistency	No serious indirectness	Very serious ^1^	None	4/92 (4.3%)	10/88 (11.4%)	RR 0.44 (0.15 to 1.33)	64 fewer per 1000, from 97 fewer to 38 more	LOW	Critical
Mortality												
3	Randomized trials	No serious risk of bias	No serious inconsistency	No serious indirectness	Very serious ^1^	None	3/92 (3.3%)	5/88 (5.7%)	OR 0.73 (0.32 to 1.68)	15 fewer per 1000, from 38 fewer to 35 more	LOW	Critical

1—Very serious imprecision due to the small sample size, fewer than 200 participants. 2—Very serious inconsistency due to substantial heterogeneity, I^2^ > 75%. Abbreviations: CI, confidence interval; MD, mean difference; OR, odds ratio; PCR, polymerase chain reaction; RoB, risk of bias; RR, risk ratio.

**Table A3:** Effect of ozone therapy for diabetic foot ulcers.

Quality Assessment							No of Patients		Effect		Quality	Importance
No of Studies	Design	RoB	Inconsistency	Indirectness	Imprecision	Other Considerations	Ozone Therapy	Control	Relative (95% CI)	Absolute		
Ulcers healed												
2	Randomized trials	Serious ^2^	No serious inconsistency	No serious indirectness	Serious ^1^	None	19/56 (33.9%)	11/55 (20.0%)	RR 1.69 (0.90 to 3.17)	138 more per 1000, from 20 fewer to 434 more	LOW	Critical
Reduction in ulcer area												
2	Randomized trials	Serious ^2^	Very serious ^3^	No serious indirectness	Serious ^1^	None	56	55	-	MD 2.11 lower, 95% CI 5.29 lower to 1.07 higher	VERY LOW	Important

1—Small sample size, fewer than 200 participants. 2—Risk of bias concerns in the included trials. 3—Very serious inconsistency due to substantial heterogeneity, I^2^ = 94.14%. Abbreviations: CI, confidence interval; MD, mean difference; RoB, risk of bias; RR, risk ratio.

**Table A4:** Effect of ozone therapy in patients who underwent surgery for impacted mandibular third molar.

Quality Assessment							No of Patients		Effect		Quality	Importance
No of Studies	Design	Risk of Bias (RoB)	Inconsistency	Indirectness	Imprecision	Other Considerations	Ozone Therapy	Control	Relative (95% CI)	Absolute		
Swelling at 72 h												
4	Randomized trials	Very serious ^1^	Very serious ^2^	No serious indirectness	Serious ^3^	None	133	133	-	SMD 0.63 higher, 95% CI 1.42 lower to 2.69 higher	VERY LOW	Important
Swelling at 7 days												
4	Randomized trials	Very serious ^1^	Very serious ^2^	No serious indirectness	Serious ^3^	None	133	133	-	SMD 0.87 lower, 95% CI 2.31 lower to 0.56 higher	VERY LOW	Important
Mouth opening at 24 h												
4	Randomized trials	Very serious ^1^	Very serious ^2^	No serious indirectness	Serious ^3^	None	133	133	-	MD 2.74 higher, 95% CI 1.93 lower to 7.41 higher	VERY LOW	Important
Mouth opening at 72 h												
4	Randomized trials	Very serious ^1^	Very serious ^2^	No serious indirectness	Serious ^3^	None	133	133	-	MD 2.77 higher, 95% CI 0.63 lower to 6.17 higher	VERY LOW	Important
Mouth opening at 7 days												
4	Randomized trials	Very serious ^1^	Very serious ^2^	No serious indirectness	Serious ^3^	None	133	133	-	MD 1.42 higher, 95% CI 1.34 lower to 4.18 higher	VERY LOW	Important
Quality of life at 7 days												
2	Randomized trials	Very serious ^1^	No serious inconsistency	No serious indirectness	Very serious ^4^	None	80	80	-	MD 6.85 lower, 95% CI 7.68 lower to 6.02 lower	VERY LOW	Critical
Quality of life at 72 h												
2	Randomized trials	Very serious ^1^	No serious inconsistency	No serious indirectness	Very serious ^4^	None	80	80	-	MD 11.08 lower, 95% CI 12.08 lower to 10.08 lower	VERY LOW	Critical
Quality of life at 24 h												
2	Randomized trials	Very serious ^1^	No serious inconsistency	No serious indirectness	Very serious ^4^	None	80	80	-	MD 11.24 lower, 95% CI 12.73 lower to 9.75 lower	VERY LOW	Critical
Number of analgesics used												
3	Randomized trials	Very serious ^1^	No serious inconsistency	No serious indirectness	Serious ^3^	None	113	113	-	MD 3.80 lower, 95% CI 4.38 lower to 3.22 lower	VERY LOW	Important

1—RoB present in >66% of included trials. 2—I^2^ > 75%. 3—Sample size between 200 and 400. 4—Sample size < 200. Abbreviations: CI, confidence interval; MD, mean difference; RoB, risk of bias; SMD, standardized mean difference.
