# Supplementary material for: Effectiveness and Safety of Ozone Therapy in Humans: An Umbrella Review of Systematic Reviews with Meta-Analyses of Randomized Clinical Trials
Source: Med Sci (Basel). 2026 Jun 4;14(2):289. doi: 10.3390/medsci14020289 (PMC13303763; doi:10.3390/medsci14020289)
Supplement: Supplementary file 1 [file medsci-14-00289-s001.zip › medsci-4291493-supplementary.pdf]

Supplementary Table S1. Database-specific search strategies used for the identification of eligible studies.

Database	Search strategy	#
Original search		
Pubmed	(("Ozone therapy"[Title/Abstract] OR "ozone treatment"[Title/Abstract] OR "ozone"[Title/Abstract])) AND (("meta-analysis"[Publication Type] OR "systematic review"[Title/Abstract]))	389
Web of Science	TS=("Ozone therapy" OR "ozone treatment" OR "ozone") AND TS=("meta-analysis" OR "systematic review")	646
Embase	('ozone therapy'/exp OR 'ozone treatment' OR 'ozone') AND ('meta-analysis'/exp OR 'systematic review/exp')	274
Cochrane Central	("Ozone therapy" OR "ozone treatment" OR "ozone") AND ("meta-analysis" OR "systematic review")	3
May 9th, 2026 update		
Pubmed	(("Ozone therapy"[Title/Abstract] OR "ozone treatment"[Title/Abstract] OR "ozone"[Title/Abstract])) AND (("meta-analysis"[Publication Type] OR "systematic review"[Title/Abstract]))	73
Web of Science	TS=("Ozone therapy" OR "ozone treatment" OR "ozone") AND TS=("meta-analysis" OR "systematic review")	141
Embase	('ozone therapy'/exp OR 'ozone treatment' OR 'ozone') AND ('meta-analysis'/exp OR 'systematic review/exp')	84
Cochrane Central	("Ozone therapy" OR "ozone treatment" OR "ozone") AND ("meta-analysis" OR "systematic review")	0

Supplementary Table S2. List of excluded studies with reasons for exclusion at full-text screening.

Study	Exclusion reason
**Alimohammadi 2025**	Wrong outcome
**Al-Moraissi 2022**	Wrong study design
**Andrade 2019**	Wrong comparator
**Anil 2021**	Wrong study design
**Arias-Vázquez 2024**	Wrong comparator
**Arias-Vázquez 2019**	Full text only available in spanish language
**Arias-Vázquez 2019**	Wrong comparator
**Arias-Vázquez 2019**	Wrong study design
**Arias-Vázquez 2026**	Wrong comparator
**Azarpazhooh 2008**	Wrong study design
**Badhe 2022**	Wrong study design
**Bansode 2024**	Wrong study design
**Bomfim 2021**	Wrong study design
**Brazzelli 2006**	Wrong study design
**Budi 2022**	Doubled
**Cao 2025**	Wrong comparator
**Carlos 2024**	Wrong study design
**Carmona 2006**	Full text only available in spanish language
**Chalidis 2023**	Wrong study design
**Chang 2024**	Wrong comparator
**Contreras 2024**	Full text only available in spanish language
**Costa 2018**	Wrong study design
**Costa 2018**	Full text only available in Portuguese language
**D'Ambrosio 2023**	Wrong study design
**Daste 2021**	Wrong comparator
**DeOliveiraMagalhaes 2012**	Wrong comparator
**Deepthi 2020**	Wrong comparator
**DiFede 2021**	Wrong intervention
**Dietrich 2021**	Wrong comparator
**Ding 2021**	Wrong intervention
**Dou 2014**	Full text not available
**Dziadek 2024**	Wrong study design
**Falci 2024**	Wrong study design
**FerreiraJr 2021**	Full text not available
**Firoozi 2022**	No RCTs evaluated
**Fitzpatrick 2018**	Wrong comparator
**Fliefel 2015**	Wrong study design
**Gc 2022**	Wrong study design
**Ghatge 2024**	No RCTs evaluated
**Giurazza 2017**	Wrong study design
**Goker 2021**	Wrong study design
**Hedayatabad 2020**	Wrong comparator
**Huang 2019**	Wrong study design

**Inchingolo 2023**	Wrong study design
**Izadi 2024**	Full text not available
**Liao 2023**	Wrong study design
**Liaqat 2023**	Wrong study design
**Lin 2022**	Wrong comparator
**Lino 2024**	Wrong study design
**Llombart-Blanco 2024**	Wrong comparator
**Magalhaes 2012**	Wrong comparator
**Maglia 2024**	Wrong study design
**Marotta 2023**	Wrong study design
**Meire 2023**	Wrong comparator
**Meng 2022**	Wrong study design
**Migliorini 2025**	Wrong comparator
**Migliorini 2020**	Wrong comparator
**Mola 2024**	Conference abstract
**Naja 2021**	Wrong study design
**Noori-Zadeh 2019**	Wrong comparator
**OliveiraModena 2022**	Wrong study design
**Palma 2023**	Wrong study design
**Pardo 2025**	Wrong comparator
**Pellicanò 2011**	Wrong study design
**Raeissadat 2018**	Wrong comparator
**Raeissadat 2018**	Wrong comparator
**Rahimzadeh 2022**	Wrong comparator
**Randi 2024**	Wrong study design
**Rappazzo 2021**	Wrong setting
**Rimeika 2021**	Wrong intervention
**Romary 2023**	Human and Animal subjects
**Sacco 2019**	Wrong study design
**Santos 2020**	Wrong comparator
**Sconza 2021**	Wrong study design
**Sconza 2020**	Wrong study design
**Serafini 2023**	Wrong study design
**Shah 2024**	Wrong study design
**Silva 2020**	Wrong study design
**Staal 2008**	Wrong study design
**Staal 2009**	Wrong study design
**Steppan 2010**	Wrong comparator
**Sun 2022**	Wrong intervention
**Tedesco 2022**	Wrong study design
**Torres-Rosas 2023**	Wrong comparator
**Veneri 2024**	Wrong study design
**Viebahn-Haensler 2024**	Wrong study design
**Wang 2023**	Wrong intervention
**Wen 2020**	Wrong study design

**Wen 2022**	Wrong comparator
**Xue 2023**	Wrong study design
**Yang 2024**	Wrong comparator
**Zanatta 2024**	Wrong intervention

Supplementary Table S3. AMSTAR 2 quality assessment of included systematic reviews and meta-analyses.

Author, Year	1	2 [b]	3	4 [b]	5	6	7 [b]	8	9 [b]	10	11 [b]	12	13 [b]	14	15 [b]	16	Overall rating
Chaundri et al., 2021	Yes	No	Yes	Partial Yes	Yes	Yes	No	Yes	Yes	No	Yes	Yes	Yes	Yes	No	Yes	**CRITICALLY LOW**
Liu et al., 2015	No	Yes	Yes	Partial Yes	No	Yes	No	Partial Yes	Yes	Yes	Yes	Yes	Yes	Yes	Yes	No	**HIGH**
Moraschini et al., 2020	Yes	No	No	Partial Yes	Yes	Yes	No	Yes	Yes	No	Yes	No	No	No	Yes	Yes	**LOW**
Liu et al., 2025	Yes	No	No	Partial Yes	No	Yes	No	Yes	Yes	No	No	No	Yes	No	No	Yes	**CRITICALLY LOW**
Shang et al., 2023	Yes	Yes	No	Partial Yes	Yes	Yes	No	Partial Yes	Yes	No	Yes	No	Yes	Yes	Yes	Yes	**LOW**
Filho et al., 2024	Yes	Yes	No	Partial Yes	Yes	No	No	Yes	Yes	No	Yes	No	Yes	Yes	Yes	Yes	**LOW**
Jafari-Oori et al., 2022	No	Yes	Yes	Partial Yes	No	Yes	No	Partial Yes	Yes	No	Yes	Yes	Yes	Yes	Yes	No	**HIGH**

Notes:

1. **Did the research questions and inclusion criteria for the review include the components of PICO** (Population, Intervention, Comparator group, Outcome)? YES/NO. For yes, must have all four.
2. **Did the report of the review contain an explicit statement that the review methods were established prior to the conduct of the review and did the report justify any significant deviations from the protocol?** YES, PARTIAL YES, NO. For Partial YES: the authors state that they had a written protocol or guide that included ALL the following (review question(s), a search strategy, inclusion/exclusion criteria, a risk of bias assessment). For YES: as for partial yes, plus the protocol should be registered and should also have specified: a meta-analysis/synthesis plan, if appropriate, and a plan for investigating causes of heterogeneity, justification for any deviations from the protocol.

3. **Did the review authors explain their selection of the study designs for inclusion in the review?** YES/NO. For YES, the review should satisfy one of the following: explanation for including only RCTs, or explanation for including only NRSI, or explanation for including both RCTs and NRSI.
4. **Did the review authors use a comprehensive literature search strategy?** YES, PARTIAL YES, NO. for PARTIAL YES must have all of the following: searched at least 2 databases (relevant to research question), provided key word and/or search strategy, justified publication restrictions (eg. Language). For YES should also have all of the following: searched the reference lists/biographies of included studies, searched trial/study registries, included/consulted content experts in the field, searched for grey literature where relevant, conducted search within 24 months of completion of the review.
5. **Did the review authors perform study selection in duplicate?** YES/NO. for YES, either ONE of the following: at least two reviewers independently agreed on selection of eligible studies and achieved consensus on which studies to include OR two reviewers selected a sample of eligible studies and achieved good agreement (at least 80 per cent) with the remainder selected by one reviewer.
6. **Did the review authors perform data extraction in duplicate?** YES/NO. For YES, either one of the following: at least two reviewers achieved consensus on which data to extract from included studies OR two reviewers extracted data from a sample of eligible studies and achieved good agreement (at least 80 per cent) with the remainder extracted by one reviewer.
7. **Did the review authors provide a list of excluded studies to justify the exclusions?** YES, PARTIAL YES, NO. FOR partial yes must provide a list of all potentially relevant studies that were read in full text form but excluded from the review. For YES must also have justified the exclusion from the review of each potentially relevant study.
8. **Did the review authors describe the included studies in adequate detail?** YES, PARTIAL YES, NO. For PARTIAL YES, must describe all of the following: populations, interventions, comparators, outcomes, research designs. For YES should also have all of the following: described populations in detail, described intervention and comparator in detail (including doses where relevant), described study setting, timeframe or follow-up.
9. **Did the review authors use a satisfactory technique for assessing the risk of bias (RoB) in individual studies that were included in the review?** For RCTs: YES, PARTIAL YES, NO, INCLUDES ONLY NRSI. For PARTIAL YES must have assessed RoB from unconcealed allocation and lack of blinding of patients and assessors when assessing outcomes (unnecessary for objective outcomes such as all cause mortality); for YES must also have assessed RoB from allocation sequence that was not truly random and selection of the reported result from among multiple measurements or analyses of a specified outcome. For NRSI (Non Randomized Studies of Intervention): YES, PARTIAL YES, NO, INCLUDES ONLY RCTs. For PARTIAL YES must have assessed RoB from confounding and from selection bias. For YES, must also have assessed methods used to ascertain exposures and outcomes, and selection of the reported results from among multiple measurements or analyses of a specified outcome.
10. **Did the review authors report on the sources of funding for the studies included in the review?** YES/NO. For YES: must have reported on the sources of funding for individual studies included in the review. Note: reporting that the reviewers looked for this information but it was not reported by study authors also qualifies
11. **If meta-analysis was performed, did the review authors use appropriate methods for statistical combination of results?** For RCTs: YES, NO, NO META-ANALYSIS. For YES: the authors justified combining the data in a meta-analysis and they used an appropriate weighted

technique to combine study results and adjusted for heterogeneity if present and investigated the causes of heterogeneity. For NRSI: YES, NO, NO META-ANALYSIS CONDUCTED. For YES: the authors justified combining the data in a meta-analysis and they used an appropriate weighted technique to combine study results, adjusting for heterogeneity if present, and they statistically combined effects estimates from NRSI that were adjusted for confounding, rather than combining raw data, or justified combining raw data when adjusted effect estimates were not available, and they reported separate summary estimates for RCTs and NRSI separately when both were included in the review.

12. **If meta-analysis was performed, did the review authors assess the potential impact of RoB in individual studies on the results of the meta-analysis or other evidence synthesis?** YES, NO, NO META-ANALYSIS INCLUDED. For YES: included only low risk of bias RCTs or, if the pooled estimate was based on RCTs and/or NRSI at variable RoB, the authors performed analysis ton investigate possible impact of RoB on summary estimates of effect.
13. **Did the review authors account for RoB in individual studies when interpreting/discussing the results of the review?** YES/NO. for YES: included only low risk of bias RCTs or, if RCTs with moderate or high RoB, or NRSI were included, the review provided a discussion of the key impact of RoB on the results
14. **Did the review authors provide a satisfactory explanation for, and discussion of, any heterogeneity observed in the results of the review?** YES/NO. For Yes: there was no significant heterogeneity in the results OR if heterogeneity was present the authors performed an investigation of sources of any heterogeneity in the results and discussed the impact of this on the results of the review
15. **If they performed quantitative synthesis did the review authors carry out an adequate investigation of publication bias (small study bias) and discuss its likely impact on the results of the review?** YES, NO, NO META-ANALYSIS CONDUCTED. For YES: performed graphical statistical tests for publication bias and discussed the likelihood and magnitude of impact of publication bias
16. **Did the review authors report any potential sources of conflict of interest, including any funding they received for conducting the review?** YES/NO. For Yes: the authors reported no competing interests OR the authors described their funding sources and how they managed potential conflicts of interest.

Rating overall confidence in the results of the review:

HIGH: no on one non-critical weakness: the systematic review provides an accurate and comprehensive summary of the results of the available studies that address the question of interest

MODERATE: more than one non critical weakness (multiple non-critical weaknesses may diminish confidence in the review and it may be appropriate to move the overall appraisal down from moderate to low confidence): the systematic review has more than one weakness but no critical flaws. It may provide an accurate summary of the results of the available studies that were included in the review

LOW: one critical flaw with or without non-critical weaknesses: the review has a critical flaw and may not provide an accurate and comprehensive summary of the available studies that address the question of interest

CRITICALLY LOW: more than one critical flaw with or without non-critical weaknesses: the review has more than one critical flaw and should not be relied on to provide an accurate and comprehensive summary of the available studies.
